# Compatibility of aztreonam in four commercial peritoneal dialysis fluids

**DOI:** 10.1038/s41598-020-58391-y

**Published:** 2020-02-04

**Authors:** Selma Tobudic, Isabella Prager, Manuel Kussmann, Markus Obermüller, Martin Ursli, Markus Zeitlinger, Martin Wiesholzer, Heinz Burgmann, Wolfgang Poeppl, Gottfried Reznicek

**Affiliations:** 1 Department of Internal Medicine I, Division of Infectious Diseases and Tropical Medicine, Medical University of Vienna, Vienna, Austria; 2 Department of Pharmacognosy, University of Vienna, Vienna, Austria; 3 Department of Internal Medicine I, University Hospital St. Poelten, Karl Landsteiner University of Health Sciences, St. Poelten, Austria; 4 Department of Clinical Pharmacology, Medical University Vienna, Vienna, Austria; 5 Department of Dermatology and Tropical Medicine, Military Medical Cluster East, Austrian Armed Forces, Vienna, Austria

**Keywords:** Preclinical research, Peritoneal dialysis

## Abstract

The preferable route for treatment of peritoneal dialysis related peritonitis remains the intraperitoneal administration of antibiotics admixed to peritoneal dialysis fluids. It is important to know whether the administered drug is compatible with the PD fluids and its container. In the present study the compatibility of aztreonam with four commercial PDFs at storing temperatures and duration representative for storing conditions in the clinical settings was investigated. Aztreonam concentrations were determined using high-performance liquid chromatography. The antimicrobial activity of aztreonam was evaluated using an *E. coli* diffusion disk inhibition assay and *P. aeruginosa* time-kill curves. In Extraneal evaluated at 6 °C, 25 °C and 37 °C aztreonam was stable over the whole study period of 14 days and 24 hours, respectively. In Physioneal and Nutrineal aztreonam was stable at 6 °C for up to 14 days. Antimicrobial activity was retained in all PD fluids over the whole study period. Aztreonam remained stable and was compatible with the PD fluids, particularly with Extraneal or Nutrineal, and no compensatory dose adjustment is needed when stored for up to 14 days at refrigeration temperature before use.

## Introduction

Bacterial peritonitis is a serious complication of peritoneal dialysis (PD) which often leads to membrane failure and patients discontinuing from peritoneal dialysis and switching to hemodialysis^1^. The most common bacteria isolated from patients with PD associated peritonitis (PDAP) are primarily Gram-positive cocci like Staphylococci or Streptococci, but also Gram-negative bacteria like *Escherichia coli* or *Pseudomonas aeruginosa* are frequently detected^2^. Particularly *P. aeruginosa* peritonitis is generally severe and associated with higher frequencies of hospitalization, catheter removal and transfer from PD to hemodialysis (HD)^3–5^. Thus, empiric antimicrobial therapy should cover both, the most likely Gram-positive and Gram-negative bacteria. Aztreonam is the only clinically available monobactam with its antibacterial spectrum limited to Gram-negative aerobic bacteria and could be of strong use for the treatment of Gram-negative PDAP. In case of a *P. aeruginosa* peritonitis the current International Society for Peritoneal Dialysis (ISPD) guidelines suggest treating with two antibiotic agents with different mechanisms of action for three weeks^1^. Therefore, aztreonam might be a feasible alternative to cephalosporins or fluoroquinolones, especially due increasing rate of multidrug-resistant Gram-negative bacteria^6^. Intraperitoneal (i.p.) administration of drugs is preferable to intravenous or oral routes in PDAP due to the markedly higher concentrations achieved at the target site^1^. In the past, aztreonam was assumed to be more cost intensive and more difficult to handle in continuous ambulatory peritoneal dialysis (CAPD), especially compared to fluroquinolones, since it must be administered four times daily^7,8^. However, more recent studies have shown that once a day i.p. administration provides sufficient serum levels and is effective in treating peritonitis in CAPD patients^9,10^. Newer studies on pharmacokinetics of aztreonam with more modern peritoneal dialysis fluids (PDFs) are lacking. Current peritoneal dialysis fluids (PDFs) used in clinical setting show differences regarding pH as well as type and concentration of the osmotic agent (glucose, amino acids and icodextrin) and buffer. It is important for prescriber to know whether the administered drug is compatible with the PD fluids and its container and whether the used PDF influences the activity of the antimicrobial agent^11^. There are no data concerning the stability and microbial activity of aztreonam in different PDFs. Thus, the present study set out to investigate the compatibility and microbial activity of aztreonam in glucose-, icodextrin-, and amino acid-containing commercial PDFs.

## Material and Methods

### Sample preparation

The compatibility of aztreonam was investigated in four different commercially available PDFs (Extraneal with 7.5% icodextrin, 2000 mL PVC bag; Physioneal with 1.36% glucose, 2000 mL double chamber PVC bag; Physioneal with 2.27% glucose, 2000 mL double chamber PVC bag; Nutrineal PD4 with 1.1% amino acids, 2500 mL PVC bag; all Baxter Healthcare Corp., Deerfield, IL, USA) at three different temperatures (refrigeration temperature (6 °C), room temperature (25 °C) and body temperature (37 °C). These three temperatures are usually used in similar studies and represent temperature conditions in daily clinical practice^12–14^. Deionized water (DIW) was used as control solution. In total, we used 48 PDF bags (12 bags per PDF). Three aztreonam containing bags and one control bag without study drug were used at each storage condition investigated^12^. All PDF bags were stored light-protected. Aztreonam (1000 mg dry powder, Bristol-Myers Squibb) was diluted according to the manufacturer instructions in 10 ml sterile distilled water and injected into the dialysis bags. Similar to real practice, for the compatibility evaluation at body temperature (37 °C) the two-chamber bag system of Physioneal was mixed directly after the application of aztreonam, whereas for the evaluation at 6 °C and 25 °C the two chambers remained separated. PDF samples of 1 ml were taken in duplicate at specific time points: 0 (directly after addition of aztreonam), 1, 2, 3, 7, 14 days for storage at refrigeration (6 °C) and room temperature (25 °C) and 0, 1, 4, 12, 24 hours for storage at body temperature (37 °C)^12–14^. Before each sample collection the bags were thoroughly shaken and visually inspected for color changes, particulate matter or haze. The concentration of aztreonam was determined by using stability indicating high-performance liquid chromatography (HPLC). The mean ± SD aztreonam concentrations were calculated from the samples taken from each storage condition (three bags × duplicate analysis). A mean loss of greater than 10% of the initial concentration was considered as clinically important loss of potency^12–14^.

#### *In vitro* antimicrobial activity testing

A disk diffusion inhibition assay with *E. coli* (ATCC 25922) was used to evaluate the antimicrobial activity after exposure to different storage conditions and periods^12,13^. Therefore, bacteria were grown overnight on Columbia agar plates (COS; 5% sheep blood Columbia agar plates, Biomerieux) resuspended in 0.9% sterile saline to a concentration equivalent to 0.5 McFarland standard and plated on COS agar plates. Each sampling point was evaluated in duplicates by impregnating 6 mm filter disks with respective sample. Inhibitory zone diameters were measured after an incubation of 24 hours at 37 °C (±1 °C). Obtained inhibitory zone diameters were compared to the initially measured concentrations, directly after injection of aztreonam^12^. Aqueous solutions containing equal aztreonam concentrations and blank PDFs without aztreonam were tested for quality assurance (data not shown).

Time-kill curves with *P. aeruginosa* ATCC 27853 were performed in Physioneal with 2.27% glucose, Nutrineal, Extraneal, and in cation-adjusted Mueller Hinton broth (CA-MHB) as comparator fluid^15^. Bacteria were grown overnight on 5% sheep blood agar plates at 37 °C, suspended in 0.9% sterile saline to a concentration equivalent to a 0.5 McFarland standard, and diluted 1:100 in PDFs or CA-MHB to obtain final inoculums^16^. Aztreonam was added in PDFs and CA-MHB to achieve final concentration of 20 µg/ml (5 × MIC) and incubation followed on an orbital shaker for 24 h at 37 °C. Samples were taken at 0, 2, 6, 8, and 24 h. Control assays without aztreonam were run in all PDFs and in CA-MHB comparator fluid. Bacterial counts were determined by using tenfold dilution plated on 5% sheep blood agar plates which were incubated for 24 h at 37 °C.

#### Sample analysis by high performance liquid chromatography (HPLC)

For the chemical analysis of aztreonam stability, a HPLC-system (Shimadzu LC-20 series) was employed consisting of a DGU-20A5 degasser, a LC-20AD quaternary pump, a SIL-20AC autosampler, a CBM-20A communication bus module, a SPD-M20A diode array detector and a CTO-20AC column oven, operated via Shimadzu LabSolutions software 5.57 SP1. LC separation was performed on an Acclaim 120 C18 column (3 µm, 120 Å, 150 × 2.1 mm I.D., serial number 002697, lot number 018-01-139, Thermo Fisher Scientific), preceded by an Acclaim 120 C18 guard cartridge (5 µm, 120 Å, 10 × 2 mm I.D., Thermo Fisher Scientific), at a flow rate of 0.50 mL/min and a column temperature of 25 °C^17^. The mobile phase consisted of a linear gradient mixed from 0.1% aqueous formic acid (mobile phase A) and acetonitrile with 0.1% formic acid (mobile phase B). The gradient started with 9% B to 24% B in 7.5 min, then purging with 95% B for 2.5 min. and again 9% B to equilibrate the column for 5.0 min before application of the next sample (total analysis time 15.0 min)^17^. The injection volume was 5 µl each and the detection wavelength was set to 264 nm to quantify peak areas of standards, samples and possible degradation products, aztreonam eluted at 4.45 min. No shift of the retention time could be observed at all during the complete period of analysis^17^. The quantification was carried out using a calibration curve with aztreonam as external standard. After collection the samples were analysed immediately. The autosampler tray was kept at 10 °C, preliminary investigations showed that the storage of the samples in the autosampler at 10 °C till analysis did not affect the aztreonam concentrations at all (data not shown). Each sample series was analyzed within one day, the single samples were analyzed twice (6 measurements per time point and temperature) and the mean value was calculated^17^. Each sample series was interspersed with several Quality Control (QC) samples of known concentrations to ensure the validity of the results. Aztreonam gave an isolated peak in the chromatogram with nice peak shape (symmetry factor 1.0 to 1.15). In the present study C-18 material was used for HPLC analysis with particle size of 3 µm achieving an excellent separation and peak shape. Only very small additional peaks could be detected in any of the sample solutions that could be separated very well from the aztreonam peak. Via PDA using other non-selective wavelength (e.g. 190 nm), all chromatograms were re-evaluated, no further peaks, peak shoulders or similar phenomena could be observed, indicating no (co)eluting other compound(s). In addition, the peak purity of the aztreonam peak was checked for all analyses via PDA. Peak purity analysis is designed to detect the presence of impurities coeluting with the analyte peak. This peak purity analysis is based on the comparison of the various UV-spectra recorded during the elution of the peak (e.g. starting point, upslope, apex, downslope, end point). If the peak is pure, then, apart from concentration differences, the spectra taken at several points during a peak should all be identical, and the match scores obtained (range from 0 to 1.0000) should be very close to the perfect match. Thus, coeluting (degradation) products would lead to significant deviations and poor match scores. In this study peak purities of 0.9999 were found for the aztreonam peaks (range from 0 to 1.0000) attesting an excellent peak purity and thus coeluting degradation products can be excluded^17^. System suitability test of the standard solution gave 0.45% RSD (peak area, n = 6). To test the specificity of the method, the single PDFs were injected, and no peak could be detected within the detection window of aztreonam. Calculated via PDA detector, no carry over could be seen after serial injection (10 times) of the standard solution and the aztreonam peak showed a peak purity of 0.9999. The precision of the method (aztreonam solution in PDF) gave 0.71% RSD (peak area, n = 6) indicating superior repeatability of the procedure. The linearity of the standard calibration curve between 5 mg/L and 1500 mg/L (corresponding to 1% to 300% of the expected aztreonam concentrations) showed r = 0.99996. The concentrations are exhibited as mean values (3 PD bags each analysed twice at the same time point and temperature).

## Results

### Physical and chemical compatibility testing

During the entire study at all three temperatures there was no visual evidence of aztreonam precipitation or color change. Analysis of HPLC data, shown in Table 1, presents the relative concentrations of aztreonam compared to the respective baseline of the four different PDFs. Aztreonam was stable in Extraneal at all temperature conditions evaluated over the entire study period of 14 days and 24 hours respectively. It was less stable in Physioneal and Nutrineal at 25 °C with ≥10% reduction of aztreonam concentration detected already after 7 days. In Physioneal with 2.27% glucose, ≥10% loss of aztreonam concentration was detected at 37 °C after 4 h.

**Table 1:** Percentage of the calculated target concentration of aztreonam (mean percentage + /- standard deviation) in four different commercially available peritoneal dialysis fluids at various temperatures and time points. Temp. = temperature, d = day(s), h = hours.

Temp.	Time	Extraneal (7.5% Icodextrin)	Physioneal (1.36% Glucose)	Physioneal (2.27% Glucose)	Nutrineal PD4 (1.1% amino acids)
6 °C	0 d	99.12 ± 1.26	98.52 ± 1.44	95.60 ± 1.28	95.75 ± 2.15
	1 d	99.17 ± 0.86	97.08 ± 0.54	95.11 ± 1.12	95.26 ± 1.88
	2 d	98.8 ± 0.90	97.44 ± 0.61	94.95 ± 1.19	94.99 ± 2.06
	3 d	98.49 ± 1.19	97.70 ± 0.80	94.81 ± 1.27	94.35 ± 2.17
	7 d	99.08 ± 1.26	97.55 ± 0.66	94.74 ± 1.20	94.06 ± 2.07
	14 d	98.46 ± 1.27	96.76 ± 0.49	94.36 ± 1.22	92.68 ± 2.05
25 °C	0 d	96.26 ± 2.79	95.18 ± 1.61	94.84 ± 1.73	96.29 ± 1.28
	1 d	95.64 ± 2.87	93.83 ± 1.48	93.60 ± 1.73	95.75 ± 1.42
	2 d	95.59 ± 2.98	93.40 ± 1.57	92.86 ± 1.74	94.81 ± 1.61
	3 d	95.25 ± 2.83	92.84 ± 1.63	92.08 ± 1.80	93.76 ± 1.48
	7 d	94.19 ± 2.84	90.48 ± 1.56	89.64 ± 1.54	91.26 ± 1.37
	14 d	93.17 ± 2.95	86.83 ± 2.00	85.39 ± 1.53	87.09 ± 1.23
37 °C	0 d	97.37 ± 1.48	97.76 ± 0.92	96.36 ± 1.74	99.47 ± 3.06
	1 h	97.16 ± 1.07	95.39 ± 1.08	93.44 ± 2.61	96.87 ± 0.73
	2 h	96.96 ± 1.34	94.16 ± 2.02	84.70 ± 5.78	96.22 ± 0.68
	4 h	96.94 ± 1.38	89.72 ± 0.42	73.29 ± 6.79	95.99 ± 0.51
	12 h	96.54 ± 1.36	89.53 ± 0.75	83.44 ± 0.95	95.39 ± 0.68
	24 h	96.24 ± 1.26	81.2 ± 0.55	74.18 ± 1.74	94.69 ± 0.52

### *In vitro* antimicrobial testing

The *E. coli* diffusion disk inhibition assay pointed the antimicrobial activity of aztreonam with relative inhibitory zone diameters ranging from 93.4 to 107.4% of the calculated target concentrations in aqueous solution (Table 2).

**Table 2:** Inhibitory zone diameters of the *Escherichia coli* inhibition assay in % of calculated target concentrations (mean percentage +/− standard deviation) in different commercial peritoneal dialysis fluids at various temperatures and time points. Temp. = temperature, % RSAD = percentage relative standard deviation, d = day(s), h = hours.

Temp.	Time	Extraneal (7.5% Icodextrin)	Physioneal (1.36% Glucose)	Physioneal (2.27% Glucose)	Nutrineal PD4 (1.1% amino acids)
6 °C	0 d	100.0 ± 0.0	100.0 ± 0.0	100.0 ± 0.0	100.0 ± 0.0
	1 d	101.0 ± 0.5	100.0 ± 0.0	98.9 ± 3.6	100.4 ± 0.8
	2 d	99.3 ± 0.2	100.9 ± 1.2	101.8 ± 2.4	99.6 ± 0.5
	3 d	101.4 ± 0.1	98.5 ± 3.6	97.1 ± 0.9	98.6 ± 1.1
	7 d	99.5 ± 0.5	99.4 ± 4.8	100.6 ± 1.2	108.8 ± 2.5
	14 d	99.2 ± 0.1	97.3 ± 1.2	100.5 ± 2.7	109.2 ± 3.5
25 °C	0 d	100.0 ± 0.0	100.0 ± 0.0	100.0 ± 0.0	100.0 ± 0.0
	1 d	99.8 ± 1.5	102.6 ± 2.6	98.1 ± 3.3	101.5 ± 4.2
	2 d	99.5 ± 1.2	104.0 ± 6.6	99.7 ± 3.9	99.4 ± 5.2
	3 d	100.1 ± 0.4	103.9 ± 5.4	99.1 ± 5.6	107.4 ± 7.6
	7 d	100.4 ± 0.2	102.2 ± 5.2	94.4 ± 0.5	99.1 ± 12.2
	14 d	100.3 ± 0.4	100.6 ± 6.2	93.4 ± 0.0	92.6 ± 6.2
37 °C	0 d	100.0 ± 0.0	100.0 ± 0.0	100.0 ± 0.0	100.0 ± 0.0
	1 h	102.7 ± 3.1	95.8 ± 5.9	100.9 ± 1.3	103.6 ± 7.1
	2 h	101.8 ± 1.6	97.8 ± 0.1	102.0 ± 0.3	101.6 ± 4.7
	4 h	103.0 ± 1.6	96.0 ± 4.2	102.5 ± 1.7	103.2 ± 6.2
	12 h	101.8 ± 0.9	95.0 ± 2.9	102.1 ± 5.0	100.9 ± 1.2
	24 h	102.2 ± 1.5	97.8 ± 1.4	113.8 ± 1.5	91.4 ± 4.7

Time-kill curves assay of aztreonam revealed after an 24 h-incubation the reduction in log_10_ colony-forming units per milliliter of *P. aeruginosa* (mean values) as follows: 0.8 in CA-MHB, 1.0 in Physioneal with 2.27% glucose, 0.7 in Nutrineal, and 2.7 in Extraneal.

## Discussion

Aztreonam is a synthetic monobactam which has satisfactory stability for at least 24 h at 37 °C^18^. It is a safe and efficient drug for the therapy of peritonitis caused by Gram-negative bacteria in patients undergoing continuous ambulatory peritoneal dialysis (CAPD) based on species identification and MIC values^19^. In patients with CAPD, aztreonam achieves high concentrations in the peritoneal cavity as well as in serum and could therefore be used for therapy of PDAP and systemic infections^20^. For intraperitoneal drug administration it is imperative that the drug being applied is compatible with the PDFs used for storage and/or administration. In the present study the stability of aztreonam in PDFs containing 7.5% icodextrin, 1.36% and 2.27% glucose, and 1.1% amino acids was studied at refrigeration-temperature (6 °C), room-temperature 25 °C and body-temperature (37 °C).

We decided to investigate the stability of aztreonam in biocompatible PDFs, including neutral pH, low glucose degradation product (GDP) fluids and icodextrin for some reasons. A major reason is the increasing usage of new PDFs in western countries, mainly due to improved residual renal function and increased ultrafiltration and tempered fluid overload, when using icodextrin^15^. Furthermore, there is a trend towards lower inflow pain, when using neutral pH, low GDP PDFs^21^. However, data on the effects of newer PDFs on occurrence and severity of peritonitis, technique survival and patient survival remain conflicting^15^. As stated in the current ISPD guidelines on peritonitis in PD patients, data on the stability of various new antibiotics and PD fluids are limited. Therefore, studies in this area are urgently requested and clinicians should remain alert for new studies^1^.

Our results show, that aztreonam was stable in Extraneal at refrigeration-temperature (6 °C) and room-temperature (25 °C) over the whole study period, implicating that Extraneal with admixed aztreonam can be stored for up to 14 days. In addition, aztreonam was stable at body-temperature (37 °C) for a period of 24 hours.

In contrast to icodextrin containing PDFs, in glucose and amino-acid containing PDFs aztreonam demonstrated only limited stability, particularly when tested at room temperature and body temperature. While aztreonam remained stable at refrigeration-temperatures (6 °C) in Physioneal and Nutrineal for up to 14 days, at room-temperature (25 °C) it was only stable for up to 7 days. Nevertheless, these 7 days seem to be long enough for home-use, as patients suffering from peritonitis are supposed to be seen by a physician more often than only once a week. The stability at body-temperature is of greater clinical relevance, as the stability is reduced by more than 10% after 4 hours. A PDF bag is usually warmed up to body temperature before instillation of its content by placing the bag on a heating plate. However, according to our data aztreonam admixed Physioneal or Nutrineal PDFs should be instilled immediately after the warming process and should not be stored longer than 4 hours.

This is important information for clinical use, as previous studies have shown considerable degradation of antimicrobial drugs. For example, previous studies showed that a degradation rate of antibiotic > 10% occurred with meropenem after 14 days in Extraneal und Physioneal at 6 °C, and in all tested PDFs at 25 °C, with ciprofloxacin after 4 hours in Physioneal at 25 °C, whereas no degradation of fosfomycin could be observed over the tested time in all PDFs^12,13,22^.

Aside from its chemical and physical stability, the antimicrobial activity of aztreonam was found to be maintained as confirmed by the *E. coli* disk diffusion assay in all different PDFs and at all investigated temperatures during the whole study time.

The difference between stability data and antimicrobial activity might be explained by the method we used for the determination of aztreonam levels. The HPLC analysis is recommended method for stability studies. Stability indicating HPLC, as used in our study, detects and separates the intact drug in the presence of decomposition products. However, it is unknown whether these decomposition products have an antimicrobial activity or not.

Using a kill-curve assay we showed a bacteriostatic effect of aztreonam, in two of three PDFs. Decreased antimicrobial activity of aztreonam *in vitro*, in glucose containing Physioneal, could be explained due reduced growth of *P. aerigunosa* in this PDF. This phenomenon was already explained in our previous studies^16^.

These findings are in parts compatible to an older study by Fuiano *et al*. who reported the effectiveness of aztreonam in combination with cefuroxime for the therapy of PD-associated peritonitis. In this trial therapeutic plasma levels were only present until 16 hours after administration of 2 g aztreonam into the peritoneal cavity. Even though only 3 out of 17 patients suffered from Gram-negative peritonitis, 2 out of the 3 patients were culture-positive for *Pseudomonas aeruginosa*, where cefuroxime is ineffective^10^. Dratwa *et al*. showed safe and effective use of intraperitoneal aztreonam in patients with *P. aeruginosa*^19^.

The dosage recommendation for intermittent administration of aztreonam in the current ISPD guidelines is based on this study from 1989^10^. Newer studies using modern PDFs in CAPD are still lacking, as well as pharmacokinetic studies in APD. However, several reports have demonstrated that peritoneal clearance is greater in patients treated with APD than those treated with CAPD regimens^23^. Therefore, in our study compatibility of aztreonam was measured using the concentration of 1 g. However, stability data are of importance prior to further investigations, but pharmacokinetic data in APD patients are needed for further IP-dos recommendations of aztreonam.

One potential limitation of the present study is the variability of the measured concentrations during the study time. This phenomenon has also been noticed with other drugs. Adsorption of substance to the container material, the molecular weight of the osmotic agent icodextrin in Extraneal (higher than that for glucose or amino acids) and the low pH value might explain reversible phenomena such as micro-precipitation or complex formation^24^.

Further, we didn’t investigate the stability of aztreonam in lactate‐buffered Dianeal. Although in many countries this PDF will be replaced with other commercial PDFs, information about Dianeal including stability of drugs in this PDF are meaningful. This data are lacking and need to be performed in future studies.

In conclusion, intraperitoneal aztreonam might be used for Gram-negative coverage of inpatient and outpatient therapy of peritoneal dialysis related peritonitis and no compensatory dose modification is needed when stored for up to 14 days at refrigeration temperature before use. At room temperature, aztreonam admixed in Physioneal and Nutrineal can be stored for up to 7 days, in icodextrin aztreonam is stable for even up to 14 days. However, when warmed to body-temperature aztreonam-containing PDFs should be used immediately when a glucose-based PDF is used.
